# Clinical indicators that influence a clinician’s decision to start L-thyroxine treatment in prematurity with transient hypothyroxinemia

**DOI:** 10.1186/s13052-023-01516-6

**Published:** 2023-08-29

**Authors:** Aslan Yilmaz, Yavuz Ozer, Nesrin Kaya, Aydilek Dagdeviren Cakir, Hazal Cansu Culpan, Yildiz Perk, Mehmet Vural, Olcay Evliyaoglu

**Affiliations:** 1 Department of Neonatology, Cerrahpasa Faculty of Medicine, Istanbul University-Cerrahpasa, Kocamustafapasa, Fatih, Istanbul, 34098 Turkey; 2 Department of Pediatric Endocrinology, Cerrahpasa Faculty of Medicine, Istanbul University-Cerrahpasa, Kocamustafapasa, Fatih, Istanbul, 34098 Turkey; 3 Department of Public Health, Cerrahpasa Faculty of Medicine, Istanbul University-Cerrahpasa, Kocamustafapasa, Fatih, Istanbul, 34098 Turkey

**Keywords:** Preterm, Treatment, Thyroid, Systematic review, Severity, Incidence

## Abstract

**Background:**

Transient hypothyroxinemia of prematurity (THOP) is defined as a low level of circulating thyroxine (T4), despite low or normal thyroid-stimulating hormone (TSH) levels. Aims: We aimed to evaluate the incidence of THOP, the clinical and laboratory findings of preterm infants with this condition and the levothyroxine (L-T4) treatment.

**Methods:**

Preterm infants (*n* = 181) delivered at 24–34 weeks of gestation were evaluated by their thyroid function tests that were performed between the 10^th^ and 20^th^ days of postnatal life and interpreted according to the gestational age (GA) references. Clinical and laboratory characteristics of the patients with THOP and normal thyroid function tests were compared. Patients with THOP and treated with L-T4 were compared with the ones who were not regarding laboratory, and clinical characteristics.

**Results:**

Incidence of hypothyroxinemia of prematurity was 45.8% (*n* = 83). Euthyroidism, primary hypothyroidism, and subclinical hypothyroidism were diagnosed in 47.5% (*n* = 86), 5% (*n* = 9) and 1.7% (*n* = 3) of the patients, respectively. Mean birth weight (BW) and GA were significantly lower in the hypothyroxinemia group than in the euthyroid group (*p* < 0.001). L-T4 was started in 43% (*n* = 36) of the patients with THOP. Treatment initiation rate was 44.4% (*n* = 16) in 24–27 wk, 41.6% (*n* = 15) in 28–30 wk, and 13.8% (*n* = 5) in 31–34 wk. As the GA increased, the incidence of THOP and the rate of treatment initiation decreased (*p* < 0.001). The lowest free thyroxine (FT4) cut-off value was 0.72 ng/dl in the treated group. In addition, incidences of vancomycin + amikacin, caffeine, dopamine treatments, RDS, IVH, BPD, central catheter, FFP transfusion, and ventilator support were higher in the treated group (*P* < 0.05).

**Conclusion:**

This study revealed that prevalence of THOP increased as the GA and BW decreased. As the GA decreased, THOP patients requiring L-T4 treatment increased. Additionally, association with comorbid diseases increased the requirement of treatment.

## Background

The incidence of congenital hypothyroidism (CH) in neonates is about 1/2000 -1/4000, and it is the worldwide one of the most common causes for preventable mental retardation. Screening programs of CH have been established for early recognition of the disorder and preventing its related neurodevelopmental consequences [1, 2]. The most common form of CH is primary hypothyroidism, characterized by high thyroid-stimulating hormone (TSH) and low thyroxine (T4) levels. TSH screening is the most sensitive test for primary CH detection. However, a unique form of CH characterized by a delayed TSH elevation has been described in preterm infants. The screening program may not be able to identify CH in premature infants due to delayed TSH elevation; therefore, the European Society of Pediatric Endocrinology recommended that all preterm infants be re-screened in addition to the heel blood screening [3, 4]. Premature babies with average results at the first newborn screening should undergo a second recommended screening two to four weeks after birth [5]. It is always recommended to repeat the second screening for premature, as a category a risk of mild CH a false-negative neonatal screening result.

In response to the significant increase in TSH level after birth, serum T4 and T3 levels rise 24 h after birth in term infants and then gradually return to normal term infant ranges at 5 to 7 days of age. In contrast, in most premature infants born between 23 and 27 weeks of gestation, serum total T4 levels actually drop in the first week of life, while they remain stable at a certain level in babies born between 28 and 30 weeks of gestation, and total T4 increase in the first week is seen only in infants born at > 30 weeks of gestation [6]. Transient hypothyroxinemia of prematurity (THOP) is characterized by low levels of T4 along with low or normal TSH levels [7]. Although immaturity of the hypothalamic-pituitary-thyroid axis is thought as the leading cause of THOP, the etiology is multifactorial. The sudden interruption of placental transfer of T4, low thyroid glandular iodine stores, medications, and non-thyroidal illness are other essential contributors. Although there are various factors that contribute to the development of THOP, low birth weight and gestational week are the main causes due to physiological reasons [8]. Undoubtedly, low levels of free thyroxine (FT4) with elevated TSH are associated with poor neurodevelopmental outcomes, and treatment is necessary. However, a straightforward approach has not yet been determined for premature babies with hypothyroxinemia and normal TSH concentrations [9]. Continuing debate exists regarding whether THOP is harmful to the developing brain. While some studies show the benefit of levothyroxine (L-T4) treatment, there are also studies reporting that the treatment do not make a difference or even cause worse neurodevelopmental outcome [10]. Although treatment of hypothyroxinemia seems beneficial in newborns below 30 weeks of gestation, there is currently no consensus on this issue [11, 12].

Thus this study aimed to determine the incidence of THOP and compare clinical and laboratory findings of the patients with hypothyroxinemia and normal thyroid functions, and evaluate clinical and laboratory findings of the patients with hypothyroxinemia who were treated with L-T4 and reveal data that affect treatment decision.

## Methods

### Participants and data

This study is a retrospective cohort study. In the study, infants who were delivered at 24–34 weeks of gestation and followed in the neonatal intensive care unit of Cerrahpasa Faculty of Medicine, Istanbul University-Cerrahpasa between January 2014 and December 2019 were involved. File records of 538 cases were studied retrospectively. Regardless from the capillary TSH results, venous thyroid function tests of 181 patients which were performed between postnatal 10–20 days were included. Clinical and laboratory characteristics of the THOP and euthyroid groups were compared. THOP cases who were treated and not treated with L-T4 were compared regarding to clinical, laboratory, and short-term clinical characteristics.

The Institutional Ethics Committee of from Cerrahpasa Faculty of Medicine, Istanbul University-Cerrahpasa approved the study protocol (Date 13.04.202, approval no: 53714).

### Definitions

Thyroid function tests were evaluated according to gestational age (GA) references. The normal age-appropriate FT4 ranges were accepted as 1.45 ± 0.5 ng/dl for 24–27 wk; 1.65 ± 0.4 ng/dl for 28–30 wk; 1.98 ± 0.4 ng/dl for 31–34 wk. The normal age-appropriate TSH ranges were accepted as 3.9 ± 2.7 mU/L for 24–27 wk; 4.9 ± 11.2 mU/L for 28–30 wk; 3.8 ± 9.3 mU/L for 31–34 wk [13].

Transient hypothyroxinemia of prematurity was defined as low FT4 and low or normal TSH levels with respect to the age-appropriate reference range. While low FT4 level with high TSH level was defined as primary hypothyroidism, normal FT4 level with high TSH level was defined as subclinical hypothyroidism. Additional clinical findings were evaluated to differentiate central hypothyroidism from THOP. Microphallus, midline defects, nystagmus, hypoglycemia, prolonged jaundice, hypothalamic-pituitary developmental disorders, and low pituitary hormones were evaluated for central hypothyroidism [14, 15].

Additional recorded disorders were bronchopulmonary dysplasia (BPD), sepsis, patent ductus arteriosus (PDA), retinopathy, intraventricular haemorrhage, necrotizing enterocolitis. Bronchopulmonary dysplasia (BPD) was considered as development of concomitant parenchymal lung injury requiring treatment with oxygen > 21% and/or positive pressure at postmenstrual GA at 36 weeks or postnatal 56 days or at discharge (which one is earlier) [16]. Sepsis was defined as those with positive blood cultures and infants with clinical signs of systemic infection [17]. Echocardiography evaluation was performed by pediatric cardiologist and in our practice, patent ductus arteriosus (PDA) took into account as both clinical findings and echocardiographic measurements were used to diagnose a hemodynamically significant PDA [18]. Retinopathy of prematurity was considered to be stage III or greater according to the Papile classification and intraventricular hemorrhage to be grade 3 or greater [19, 20]. According to the BELL classification, necrotizing enterocolitis was diagnosed as stage 2 or higher [21]. Requirement of mechanical ventilation support (invasive or non-invasive) for at least 24 h was accepted as ventilation support.

Patients who needed inotropes, highly potent broad-spectrum antibiotics (such as vancomycin + amikacin), blood transfusion, and ventilator support diagnosed with sepsis, RDS, IVH were considered to have severe disease. During follow-up, time for the normalization of thyroid functions was defined as euthyroid time.

The treatment initiation algorithm was not determined for the cases, and a limit value was not defined for the TSH and FT4 results for the treatment decision. Pediatric endocrinologists made the treatment decision according to each case’s gestational age and thyroid function test results after the second check of TSH and FT4 results. The basic approach of our clinic in starting the treatment is as follows; It was determined that the cases diagnosed with hypothyroxinemia according to the blood results evaluated between the first 10^th^ and 20^th^ days after birth, were found to have continued hypothyroxinemia according to gestational week and postnatal age in the blood tests one week later and the TSH value continued to increase compared to the initial value.

### Statistical analysis

Data were statistically analyzed using SPSS 21.0 computer software (SPSS, Chicago, IL, USA). The normality of the distribution of continuous parameters was evaluated by the Kolmogorov- Smirnov and Shapiro–Wilk tests. Continuous variables with normal distribution were expressed as mean ± standard deviation and compared by Student t-test or One-way ANOVA test. Continuous variables without normal distribution were expressed as median (minimum–maximum) and compared by Mann–Whitney U test or Kruskal Wallis test. Categorical variables were presented as frequency (percentage) and compared by Chi-square test or Fisher's Exact test, where appropriate. The tests used to compare the parameters are given below the table. The cut-off value for FT4 was assessed using the ROC curve, and the sensitivity and specificity were given. A *p*-value of less than 0.05 was considered statistically significant.

## Results

Data were collected from 181 infants, of whom 52.5% (*n* = 95) were male. Mean GA, BW, and length of hospital stay were 29 ± 2.78 wk, 1424.85 ± 522.58 g, 45.2 ± 28.7 days, respectively. Thyroid function tests were euthyroid in 47.5% (*n* = 86) patients. Transient hypothyroxinemia of prematurity, primary hypothyroidism and subclinical hypothyroidism were detected in 45.8% (*n* = 83), 5% (*n* = 9) and 1.7% (*n* = 3), respectively. Three infants delivered at 24–27 GW died during follow-up due to pulmonary haemorrhage, sepsis, and intraventricular haemorrhage. The FT4 and TSH values of the patients treated with primary hypothyroidism diagnosis were 0.96 ± 0.34 ng/ dl and 27.22 ± 28.06 mU/L, respectively. A thyroid ultrasound has been performed in 4 cases, and all of these examinations were normal. The L-T4 dose range of the treatment started with the diagnosis of THOP was 5–10 mcg/kg/day, while the treatment dose was 10–15 mcg/kg/day with the diagnosis of primary hypothyroidism.

### THOP and Euthyroid patients

Characteristics of the THOP and euthyroid groups are presented in Table 1. The rate of being male was significantly higher in infants with THOP (*p* = 0.002). Mean GA and BW was significantly lower in the THOP group (29.1 ± 2.78 wk, 1241.6 ± 445.3 g, respectively) compared to the euthyroid group (31 ± 2.39 wk, 1611.3 ± 500,9 g, respectively) (*p* < 0.001). Length of hospital stay was significantly longer in THOP patients compared to the euthyroid group (*p* < 0.001). Serum FT4 and TSH levels of the two groups are shown in Table 1. As expected, serum FT4 levels were lower in the THOP group (*p* < 0.001), and there was no difference in serum TSH levels (*p* = 0.575).

**Table 1:** Laboratory and clinical characteristics of THOP group and euthyroid group

		**THOP Group**	**Eutyroid Group**	***p* value**
Participant, n		83	86	
Sex (Male)		53 (64.8)	34 (39.5)	**0.002**^**a**^
GA, wk		29 (24–34)	31 (26–34)	** < 0.001**^**b**^
BW, g		1190 (485–2560)	1624 (625–2895)	** < 0.001**^**b**^
GA (group)				** < 0.001**^**a**^
24–27 wk		26 (31.3)	11 (12.7)	
28–30 wk		28 (33.7)	20 (23.2)	
31–34 wk		29 (34.9)	55 (63.9)	
Length of hospital stay, d		48 (10–131)	31 (10–110)	** < 0.001**^**b**^
Initial laboratory measurement time, d		12.3 ± 1.95	12.5 ± 1.50	0.052^b^
Initial laboratory measurement GAA, wk		31.0 ± 2.83	33.0 ± 2.43	** < 0.001**^**b**^
Serum FT4 (ng / dl)		1.02 (0.31–1.58)	1.55 (0.97–2.54)	** < 0.001**^**b**^
Serum TSH ( mU/L)		3.92 (0.25–10.64)	3.54 (0.73–13.3)	0.586^b^
GA groups	Serum FT4 (ng/dl)	Serum TSH (mU/L)	Serum FT4 (ng/dl)	Serum TSH (mU/L)
24–27 wk	0.81 ± 0.25	4.31 (0.25 -7.82)	1.36 (0.97–2.2)	2.74 (1.57–6.91)
28–30 wk	0.97 ± 0.24	3.16 (0.99–10.60)	1.44 (1.25–2.54)	3.47 (1.14–13.3)
31–34 wk	1.13 ± 0.14	4.06 (0.88–9.08)	1.66 (1.20–1.94)	3.82 (0.73–11.10)
*p* value	< 0.001^c^,^e^	0.808^d^	0.001^d^,^f^	0.336^d^

Categorical data are given as frequency and (%). Continuous data with normal distribution are given as mean ± standard deviation. Median (min–max) was used when distribution is not normal. Bolds are statistically significant (*p* < 0.05)

*Abbreviations*: *THOP* transient hypothyroxinemia of prematurity, *GA* gestational age, *GAA* gestation-adjusted age, *BW* birth weight

^a^Chi-square test

^b^Mann Whitney U test

^c^One-way ANOVA test

^d^Kruskal Wallis test

^e^There was a significant difference between all groups

^f^There was a significant difference between; 24–27 and 31–34 wk., 28–30 and 31–34 wk

FT4 levels were significantly lower in patients with lower gestational ages in THOP and euthyroid groups (*p* < 0.001, *p* = 0.001, respectively), a similar difference was not observed in TSH (*p* = 0.808, *p* = 0.336) (Table 1).

### THOP patients and L-T4 treatment

TSH and FT4 results used in the study are the first serum values checked before treatment. Thirty of 36 patients diagnosed with THOP and started on L-T4 therapy were treated according to their initial serum TSH and FT4 results. Treatment was created in the remaining 6 cases due to the persistence of FT4 suppression in the weekly TFT follow-up. Characteristics of the L-T4 treated and L-T4 untreated groups are presented in Table 2. GA and BW were significantly lower in the L-T4 treated group (*p* < 0.001). Serum FT4 levels were lower in the treated group (*p* < 0.001), but no difference was observed in TSH levels. Length of hospital stay was longer in the treated patients compared to untreated patients (61.4 ± 26.1 and 46.1 ± 30 d, respectively) (*p* < 0.001). The duration of euthyroidism from the diagnosis of hypothyroxinemia in treated patients was shorter than in the untreated group (3.9 ± 0.9 and 5.1 ± 2.7 wk, respectively) (p: 0.020). In untreated group, FT4 levels increased as GA increased (*p* = 0.001) (Table 2). No such difference was observed in the treated group.

**Table 2:** Laboratory and clinical characteristics of L-T4 treated vs untreated groups of THOP patients

		**L-T4 Treated**	**L-T4 Untreated**	***p* value**
Participant, n		36 (43.3)	47 (56.6)	
Sex (Male)		23 (63.8)	30 (63.8)	0.996^a^
GA, wk		28 (24–32)	31 (24–34)	** < 0.001**^b^
BW, g		1057 ± 300	1383 ± 488	** < 0.001**^c^
SGA		8 (22)	11 (23)	0.899^a^
GA (group)				** < 0.001**^a^
24–27 wk		16 (44.4)	10 (21.2)	
28–30 wk		15 (41.6)	13 (27.6)	
31–34 wk		5 (13.8)	24 (51)	
Length of hospital stay, d		54 (21–121)	40 (10–131)	**0.003**^b^
Euthyroid time, wk		4 (3–7)	4 (3–17)	**0.020**^b^
FT4 ( ng/dl)		0.80 (0.31–1.58)	1.07 (0.73–1.44)	** < 0.001**^b^
TSH (mU/L)		3.92(0.99–10.62)	3.90 (0.25–10.64)	0.575^b^
GA groups	FT4 (ng/dl)	TSH (mU/L)	FT4 (ng/dl)	TSH (mU/L)
24–27 wk	0.74 (0.31–1.19)	5.2(1.27–7.82)	0.81(0.73–1.44)	3.42(0.25–10.64)
28–30 wk	0.91 (0.42–1.58)	3.05(0.99–7.84)	1.04(0.73–1.19)	3.47(1.25–10.6)
31–34 wk	1.06 (0.78–1.23)	3.92(2.59–7.50)	1.16(0.91–1.36)	4.11(0.88–9.08)
*p* value	0.093^d^	0.301^d^	**0.001**^d^,^e^	0.710^d^

Categorical data are given as frequency and (%). Continuous data with normal distribution are given as mean ± standard deviation. Median (min–max) was used when distribution is not normal. Bolds are statistically significant (*p* < 0.05)

*Abbreviations*: *THOP* transient hypothyroxinemia of prematurity, *GA* gestational age, *BW* birth weight

^a^Chi-square test

^b^Mann Whitney U test

^c^Student t-test

^d^Kruskal Wallis test

^e^There was a significant difference between; 24–27 and 31–34 wk., 28–30 and 31–34 wk

Incidence of medications with vancomycin + amikacin, caffeine, and dopamine, diagnosis of RDS, IVH, BPD, clinical interventions like central catheterization, fresh frozen plasma transfusion, and ventilator support was higher in the treated group (*p* < 0.05) (Table 3). However, there was no difference in the sepsis, PDA, NEC, and ROP incidences between the treated and untreated groups.

**Table 3:** Disease severity assessment of the L-T4 treated and untreated THOP patients

	**L-T4 Treated**	**L-T4 Untreated**	***p* value**^**a**^
Participants, n	36	47	
Medicine			
Vancomycin + amikacin	10 (28)	5 (11)	**0.044**
Ampicillin + gentamicin	13 (36)	26 (55)	0.082
Caffeine	34 (94)	36 (77)	**0.027**
Dobutamine	6 (17)	4 (9)	0.258
Dopamine	15 (42)	7 (15)	**0.006**
Clinical-diagnosis			
Sepsis	5 (14)	5 (11)	0.652
PDA	9 (25)	8 (17)	0.372
RDS	28 (78)	23 (49)	**0.007**
IVH	18 (50)	11 (23)	**0.022**
BPD	23 (64)	14 (30)	**0.002**
ROP	1 (3)	2 (4)	0.363
NEC	1 (3)	1 (2)	1.000
Clinical interventions			
Central catheter	35 (97)	34 (72)	**0.003**
ES transfusion	15 (42)	12 (26)	0.120
TS transfusion	7 (19)	3 (6)	0.070
FFP transfusion	18 (50)	11 (23)	**0.012**
Ventilator support	22 (61)	18 (38)	**0.039**
NIV support	24 (67)	28 (60)	0.508

Categorical data are given as frequency and (%). Bolds are statistically significant (*p* < 0.05)

When THOP cases were compared in terms of disease severity of L-T4 treated and untreated group; vancomycin + amikacin, caffeine, dopamine, RDS, IVH, BPD, central catheter, FFP transfusion, ventilator support were statistically significant (*P* < 0.05)

*Abbreviations*: *THOP* transient hypothyroxinemia of prematurity, *RDS* respiratory distress syndrome, *IVH* intraventricular haemorrhage, *BPD* bronchopulmonary dysplasia, *ROP* premature retinopathy, *NEC* necrotizing enterocolitis, *ES* erythrocyte suspension, *TS* thrombocyte suspension, *FFP* fresh frozen plasma, *NIV* non-invasive ventilation

^a^Chi-square test

**Fig. 1:**
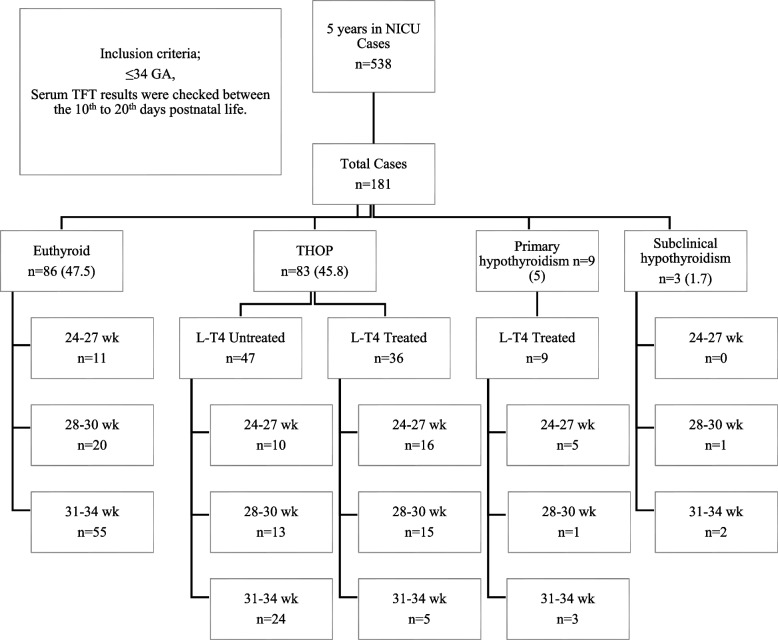
Distribution of all cases by week of gestation (data are given as numbers and %). Abbreviations: THOP, transient hypothyroxinemia of prematurity; GA, gestational age; TFT, thyroid function test

In this study, the cut-off value of FT4 for initiation of treatment was 0.72 ng/dl (specificity 100%, sensitivity 33%) (Fig. 1). L-T4 treatment was given to all THOP patients whose FT4 levels were below 0.72 ng/dl, whereas treatment was given to only 33.8% of the THOP patients whose FT4 levels were ≥ 0.72 ng/dl (24 of 71) (Table 4).

**Table 4:** GA and FT4 values of patients who were started treatment with a diagnosis of THOP

Cut-off (GA)	L-T4 Treated n (%)	L-T4 Untreated n (%)	Total n (%)
≤ 30 GA	31 (57.4)	23 (42.6)	54 (100)
> 30 GA	5 (17.2)	24 (82.8)	29 (100)
Total	36 (43.4)	47 (56.6)	83 (100)
Cut-off (FT4 = ng/dl)			
< 0.72	12 (100)	0 (0)	12 (100)
≥ 0.72	24 (33.8)	47 (66.2)	71 (100)
Total	36 (43.4)	47 (56.6)	83 (100)

Categorical data are given as frequency and (%)

## Discussion

In this cohort incidence of hypothyroxinemia of prematurity was 45.8% (*n* = 83) which increased as GA and BW decreased. There was a male predominance among patients with THOP. L-T4 treatment was started primarily in the preterm infants with lower GA and BW and whose FT4 levels were below 0.72 ng/dl. Patients with THOP had more severe disease, thus, the length of hospital stay was longer in the THOP patients compared to euthyroids. Similar to our study, the prevalence of THOP was reported to be approximately 50% in previous studies, while male gender dominance was not reported [9, 22, 23].

### THOP and Euthyroid patients

This study revealed an inverse relation between GA and the frequency of THOP and mean GA of patients with THOP was 29.1 ± 2.78 (24–34) weeks. In a recent study, mean GA of patients with hypothyroxinemia of prematurity and normal thyroid function test were determined as 26.4 ± 1.9 weeks and 28.9 ± 2.2, respectively [23]. Our recently published study was excluded gestational age, using the same patient population and ethic committee approval as this study, small for gestational age and congenital heart diseases were found to be associated factors with THOP [24]. In addition, the relationship between gestational age and the frequency of THOP has been reported in the literature [13, 25]. In this study, the relationship between the frequency of hypothyroxinemia and the gestational week was found to be compatible with the literature.

Free thyroxine levels decreased as GA decreased in the THOP and euthyroid groups. In the THOP and euthyroid groups, FT4 levels were at the lowest in infants born earlier than 28 weeks of gestation. There was no such relation between GA and serum TSH levels in both groups, and serum TSH levels were not different between THOP and euthyroid groups. The relationship between GA and T4 is well defined and has been associated with cessation of T4 transmission from the placenta to the infant [26]. Williams et al. [13] showed that the lowest postnatal FT4 level was in the group whose GA was below 27 weeks of gestation. In this and previous studies low T4 levels are not associated with TSH increments in premature newborns. Immaturity of the hypothalamic-pituitary axis in premature infants might explain low T4 being not accompanied by TSH increments, which makes the differential diagnosis of THOP, central hypothyroidism, and sick euthyroid syndrome difficult.

### THOP patients and L-T4 treatment

Mean GA, BW and FT4 level were lower in L-T4 treated patients compared to untreated. In patients with THOP frequency of L-T4 treatment increased as the GA decreased. L-T4 treatment was given mostly to the patients who were between 24–27 GA (44.4%), and the cases who were not treated mainly were in the 31–34 weeks of gestation group (51%). Before 10 to 12 weeks of gestation, the fetus depends entirely on the transplacental transmission of maternal thyroid hormones. After the 20th gestational age, fetal dependence on maternal hormones gradually decreases as the gestational age increases; however, even at term, approximately 30% of thyroid hormones measured in cord blood originate from the mother. However, preterm infants often exhibit weaker or no TSH fluctuations at birth, which can be attributed to hypothalamic-pituitary axis immaturity. Therefore, the earlier a preterm baby is born, the more inadequate thyroid hormone reserve and an underdeveloped born with the hypothalamic-pituitary axis [27, 28]. In light of the above, treatment was mostly commenced to preterm infants with lower GA in our cohort. Thyroid hormones are essential for brain development and thus should be replaced if thyroid hormone levels are low. However, this is different for premature hypothyroxinemia. Although treatment has been beneficial in some studies, some showed no effect or even worse [11, 12, 28, 29]. Thus, the initiation of treatment for THOP is controversial.

In a recent study by Sze May Ng et al. [30], premature babies born earlier than 28 weeks of gestation were divided into two groups, one group was treated with thyroxine from the first day, and the control group did not get L-T4 treatment. Motor, language, and cognitive functions were significantly higher in Bayley III development tests performed at 42 months in the group given thyroxine treatment. In an earlier study by Suzumura et al. [31] premature infants below 28 weeks of GA were evaluated in two-time intervals. In the first time interval when routine FT4 measurements were not performed 54 infants were involved who did not get LT4 treatment. In the second interval when routine T4 measurements were performed 60 premature infants who had FT4 < 0.8 ng/dl got LT4 at a dose of 5–10 µgr/kg mainly at 7 days of age. When two groups were compared at the corrected ages of 18 months and 7 days incidence of cerebral palsy was lower in the treated group. A major concern about this study is that the first interval was before the neonatal screening program, which could contribute to the results. In the study of Nomura et al. [32], treatment was initiated at a dose of 5–10 mcg/kg/day, which was found to have low FT4 on the postnatal 7th day of life, and showed that the treatment prevented neurological delay when compared with the control group. Two hundred premature infants born earlier than 30 weeks of gestation were treated with L-T4 or placebo 12–24 h after birth for 6 weeks. During hospitalization incidence of cerebral hemorrhage was higher in the treated group. At 2 years of age, 157 of them were re-evaluated and in the group born earlier than 27 gestational weeks (25–26 wk), the mean IQ was 18 points higher, whereas in the group born between 27–30 weeks mean IQ was 10 points lower in the treated groups [33]. Ten years later 113 of them were re-evaluated and school success and motor development were higher in the treated groups who were born earlier than 27 weeks of gestation (25–26 wk) and 28 weeks respectively compared to placebo groups. However, in the treatment group development was poor in premature infants born at 29 weeks of gestation and needed more special education [11]. Twenty-three infants born between 25–28 gestational weeks and with birth weight lower than 1250 gr, TT4 ≤ 4 gr/dl, and TSH ≤ 20 mU/L at the 15th day of life were treated with LT4 or placebo for 7 weeks. At the 28th and 36th weeks of life, there was no difference in PDA, NEC, retinopathy, and anthropometric measurements. Additionally, mean serum T4 levels were indifferent at 21, 35, 49, 63, and 77th days of life. Data regarding neurodevelopment was insufficient in this study [34]. In 70 premature infants with birth weight lower than 1500 g and FT4 < 0.8 ng/dl and TSH < 10mIU/ml, anthropometric measurements, cerebral palsy, and neurodevelopmental indicators were indifferent between L-T4 treated and not treated groups at the corrected age of 18 months of age [35]. In 40 infants with gestational age lower than 31 weeks, LT4 treatment did not affect neonatal mortality and morbidity, and neurodevelopment at the corrected age of 7 months (Table 5) [36].

**Table 5:** Results of the studies on the relationship between THOP and neurodevelopmental outcomes

Source and study type	**Sample size**	**Supplementary strategy and assessment age**	**Outcomes**
Sze May Ng et al. [30], randomized clinical trials	< 28 weeks of gestation, 30 infants treated, 29 infants untreated,	8 µg/kg at first day to 32 weeks of gestation, 42 months	Motor, language, and cognitive functions were significantly higher in Bayley III development tests in the group given thyroxine treatment
Suzumura et al. [31], cohort study	< 28 weeks of gestation, 54 infants (first period, untreated and not measurement), 60 infants (second period treated, FT4 < 0.8 ng/dl was)	5–10 µgr/kg mainly at 7 days of age, corrected age of 18 months	Incidence of cerebral palsy was lower in the treated group
van Wassenaer et al. [12, 33], randomized clinical trials	< 30 weeks of gestation, 100 infants treated 100 infants untreated	8 µg/kg/day at first day to 42^th^ day, 2 and 10 years	At 2 years of age, 27 gestational weeks (25–26 wk), the mean IQ was 18 points higher, whereas in the group born between 27–30 weeks mean IQ was 10 points lower in the treated groups at 2 years of age At 10 years of age school success and motor development were higher in the treated groups who were born earlier than 27 weeks of gestation (25–26 wk) and 28 weeks respectively compared to placebo groups. However, in the treatment group development was poor in premature infants born at 29 weeks of gestation and needed more special education
Chowdhry et al. [34], randomized clinical trials	25–28 weeks of gestation and birth weight < 1250 g, 11 treated 12 untreated	10 µg/kg/day, at 14 to 66 days, corrected age of 12 months	At the 28^th^ and 36^th^ weeks of life, there was no difference in PDA, NEC, retinopathy, and anthropometric measurements Data regarding neurodevelopment was insufficient in this study
Uchiyama et al. [35], randomized clinical trials	Unknown, Birth weight < 1500 g 25 infants treated, 45 infant untreated	5 µg/kg/day at 14 to 42 days, corrected age of 18 months	Anthropometric measurements, cerebral palsy, and neurodevelopmental indicators were indifferent between L-T4 treated and not treated groups
Vanhole et al. [36] randomized clinical trials	25–30 weeks of gestation, 20 treated 20 untreated	20 µg/kg/day at 1 to 14 days, age of 7 months	LT4 treatment did not affect neonatal mortality and morbidity, and neurodevelopment
Cochrane investigation [37], metaanalysis	4 study, 318 infants	LT4 treatment was started in the first 48 postnatal hours, however different doses, treatment modalities, and durations	These studies showed that prophylactic LT4 treatment did not have any effect on neonatal mortality, morbidity, and neurodevelopment
Dilli et al. [38], cohort study	< 32 weeks of gestation and < 1500 g, 16 infants were THOP, 40 infants were euthyroid	No treatment, corrected age of 18 to 24 months	THOP was not associated with increased risk of cerebral palsy or decreased mental development index and psychomotor development index scores
Hollanders et al. [22], cohort study	< 32 weeks of gestation and/or < 1500 g 120 infants were THOP, 278 infants were nonhypothyroxinemic	No treatment, 19 years of age	There was no relation between hypothyroxinemia and neurodevelopment

In a Cochrane data investigation about the effects of LT4 treatment on clinical findings and long-term development in premature infants with transient hypothyroxinemia, 4 studies were selected and a total of 318 preterm infants were evaluated. In all premature infants, LT4 treatment was started in the first 48 postnatal hours, however different doses, treatment modalities, and durations were reported. These studies showed that prophylactic LT4 treatment did not have any effect on neonatal mortality, morbidity, and neurodevelopment [37]. Additionally in another study 56 preterm infants were divided two groups (THOP and euthyroid groups) weighing 1500 g and under 32 weeks of gestation were evaluated for neurodevelopmental status at the corrected age of 18 to 24 months. After adjusting for gestational age and multiple prenatal, perinatal, and early and late neonatal variables, THOP was not associated with increased risk of cerebral palsy or decreased mental development index and psychomotor development index scores (Table 5) [38].

Reports about the effects of premature hypothyroxinemia on clinical and neurocognitive outcomes during newborn and childhood periods are controversial. In a 19-year follow-up study evaluating neurocognitive effects of hypothyroxinemia; 398 preterm infant (< 32 wk) and/or very low birth weight (< 1500 g) infants were evaluated at 19 years of age and there was no relation between hypothyroxinemia and neurodevelopment (Table 5) [26]. However, in rats, maternal T4 is needed for fetal cerebral cortex formation even in the last stages of pregnancy [39]. Although in humans in the second half of pregnancy fetal thyroid axis is effective, in premature infants early cessation of maternal T4 passage can have some contribution. The gestational week and birth weight of the cases in the L-T4 treated group in our study were lower than those of the L-T4 untreated group. For this reason, it is an expected result that the cases in the L-T4 treated group have a higher probability of having nonthyroidal illness syndrome, but we could not make this distinction in this study.

In THOP patients, incidence of drug treatments (such as vancomycin + amikacin, caffeine, and dopamine), severe additional disorders (such as RDS, IVH, BPD), and clinical interventions (such as central catheterization, ventilator support, fresh frozen plasma transfusion) were higher in the L-T4 treated group. Thus, duration of hospitalisation was longer in the treated group. Which suggests LT4 treated patients with THOP had more severe disorders compared to the untreated. Some studies showed that FT4 suppression took longer in preterm infants diagnosed with RDS [40, 41]. In our study, most of the patients in whom L-T4 treatment was initiated were preterm infants diagnosed with RDS. We observed that although the gestational age increased, sick preterm infants could not increase the FT4 levels appropriately compared to those without the additional disease. In addition, the treated group consisted of patients with a smaller gestational age and birth weight than untreated group, so it is an expected result of the need for more potent antibiotic and inotropic support treatment. This result may indicate that only premature infants with low gestational age and low birth weight are treated. FT4 levels were lower in 24–27 and 28–30 weeks compared to 31–34 weeks in untreated group whereas there was no such difference in the L-T4 treated group. Additionally period for euthyroidism was longer in untreated group, which suggests treatment was efficient in normalisation of thyroid functions.

This study showed no difference in sepsis, NEC, and ROP incidences between the treated and untreated groups. Similarly, no difference in incidences of ROP, BPD, and NEC were reported [22, 42]. Whether the underlying disorders causing hypothyroxinemia or treatment with L-T4 is beneficial remains unanswered. The lower limit of FT4 to start treatment is detected as 0.72 ng/dl in this study. There is no consensus about the treatment for patients with THOP and there is no value at which treatment should be initiated [27, 28, 43]. As intended, the meantime for achieving euthyroidism was shorter in patients who were treated than the untreated ones. Preterm infants in the treatment group stayed in the hospital longer, and incidence of BPD was higher. This study can not give the answer whether this is due to infants being more severely ill and needed more frequent respiratory support or had previously received L-T4 treatment.

The limitation of this study is its retrospective design and single-center study. The fact that the changes in the vital signs and weaning from inotropes and mechanical ventilation of the patients who received and did not receive treatment were not included in this study is another limiting factor. Another limitation is that we could not give a clear response to the clinical benefits of providing early euthyroidism in preterm infants, since randomization was not performed in this study. The strength of this study is the relatively large cohort.

## Conclusion

In conclusion, the management of THOP and the necessity of L-T4 therapy are controversial (Table 5). This study showed that the frequency of THOP and the incidence of starting L-T4 therapy increased as GA and BW decreased. Disease severity was inversely proportional to serum FT4 levels independent of GA and therefore influenced treatment decisions. We suppose that the FT4 level (especially < 0.72 ng/dl) measured between the postnatal 10^th^ and 20^th^ days in preterm infants under 34 weeks of gestation may be effective in the decision to start treatment, but we cannot draw an explicit conclusion regarding the improvement of short-term results because randomization was not performed in our study. However, comprehensive prospective studies are needed to determine the characteristics of THOP and clarify treatment strategies, including optimal dose and timing of initiation. Therefore, new trials are required to further investigate the benefits of thyroid hormones given to very preterm infants during the neonatal period.

## Data Availability

The datasets generated and/or analysed during the current study are not publicly available due to our hospital policy but are available from the corresponding author on reasonable request.

## Abbreviations

THOP: Transient hypothyroxinemia of prematurity
TFT: Thyroid function test
GA: Gestational age
GAA: Gestation-adjusted age
BW: Birth weight
RDS: Respiratory distress syndrome
IVH: Intraventricular haemorrhage
BPD: Bronchopulmonary dysplasia
ROP: Premature retinopathy
NEC: Necrotizing enterocolitis
ES: Erythrocyte suspension
TS: Thrombocyte suspension
FFP: Fresh frozen plasma
NIV: Non-invasive ventilation
