# Correction: Clinical indicators that influence a clinician’s decision to start L-thyroxine treatment in prematurity with transient hypothyroxinemia

**DOI:** 10.1186/s13052-023-01539-z

**Published:** 2023-09-29

**Authors:** Aslan Yilmaz, Yavuz Ozer, Nesrin Kaya, Aydilek Dagdeviren Cakir, Hazal Cansu Culpan, Yildiz Perk, Mehmet Vural, Olcay Evliyaoglu

**Affiliations:** 1 Department of Neonatology, Cerrahpasa Faculty of Medicine, Istanbul University-Cerrahpasa, 34098 Kocamustafapasa, Istanbul, Fatih Turkey; 2 Department of Pediatric Endocrinology, Cerrahpasa Faculty of Medicine, Istanbul University-Cerrahpasa, 34098 Kocamustafapasa, Istanbul, Fatih Turkey; 3 Department of Public Health, Cerrahpasa Faculty of Medicine, Istanbul University-Cerrahpasa, 34098 Kocamustafapasa, Istanbul, Fatih Turkey

**Italian Journal of Pediatrics (2023) 49:105**

10.1186/s13052-023-01516-6

The original article [1] contained an error in Table 2. Yilmaz et al. apologize for the mistake made while editing Table 2. This error appears correct in the results section of the original article.

The correct version of Table 2 has been included in this correction.

**Table 2:** Laboratory and Clinical Characteristics of L-T4 treated vs untreated groups of THOP patients

		L-T4 Treated	L-T4 Untreated	*p* value
Participant, n		36 (43.3)	47 (56.6)	
Sex (Male)		23 (63.8)	30 (63.8)	0.996^a^
GA, wk		28 (24–32)	31 (24–34)	**< 0.001** ^b^
BW, g		1057 ± 300	1383 ± 488	**< 0.001** ^c^
SGA		8 (22)	11 (23)	0.899^a^
GA (group)				**< 0.001** ^a^
24–27 wk		16 (44.4)	10 (21.2)	
28–30 wk		15 (41.6)	13 (27.6)	
31–34 wk		5 (13.8)	24 (51)	
Length of hospital stay, d		54 (21–121)	40 (10–131)	**0.003** ^b^
Euthyroid time, wk		3.9 ± 0.9	5.1 ± 2.7	**0.020** ^b^
FT4 ( ng/dl)		0.80 (0.31–1.58)	1.07 (0.73–1.44)	**< 0.001** ^b^
TSH (mU/L)		3.92 (0.99–10.62)	3.90 (0.25–10.64)	0.575^b^
GA groups	FT4 (ng/dl)	TSH (mU/L)	FT4 (ng/dl)	TSH (mU/L)
24–27 wk	0.74 (0.31–1.19)	5.2 (1.27–7.82)	0.81 (0.73–1.44)	3.42 (0.25–10.64)
28–30 wk	0.91 (0.42–1.58)	3.05 (0.99–7.84)	1.04 (0.73–1.19)	3.47 (1.25–10.6)
31–34 wk	1.06 (0.78–1.23)	3.92 (2.59–7.50)	1.16 (0.91–1.36)	4.11 (0.88–9.08)
*p* value	0.093^d^	0.301^d^	**0.001**^d,^*	0.710^d^

Categorical data are given as frequency and (%). Continuous data with normal distribution are given as mean ± standard deviation. Median (min-max) was used when distribution is not normal. Bolds are statistically significant (p < 0.05)

^a^ Chi-square test, ^b^ Mann Whitney U test, ^c^ Student t-test, ^d^ Kruskal Wallis test

* There was a significant difference between; 24–27 and 31–34 wk., 28–30 and 31–34 wk

Abbreviations: THOP, transient hypothyroxinemia of prematurity; GA, gestational age; BW, birth weight
